# Desmosomes in Cell Fate Determination: From Cardiogenesis to Cardiomyopathy

**DOI:** 10.3390/cells12172122

**Published:** 2023-08-22

**Authors:** Hoda Moazzen, Mistura Dolapo Bolaji, Rudolf E. Leube

**Affiliations:** Institute of Molecular and Cellular Anatomy, RWTH Aachen University, Wendlingweg 2, 52074 Aachen, Germany; mbolaji@ukaachen.de (M.D.B.); rleube@ukaachen.de (R.E.L.)

**Keywords:** desmosome, cardiogenesis, cell fate determination, epithelial to mesenchymal transition, arrhythmogenic cardiomyopathy

## Abstract

Desmosomes play a vital role in providing structural integrity to tissues that experience significant mechanical tension, including the heart. Deficiencies in desmosomal proteins lead to the development of arrhythmogenic cardiomyopathy (AC). The limited availability of preventative measures in clinical settings underscores the pressing need to gain a comprehensive understanding of desmosomal proteins not only in cardiomyocytes but also in non-myocyte residents of the heart, as they actively contribute to the progression of cardiomyopathy. This review focuses specifically on the impact of desmosome deficiency on epi- and endocardial cells. We highlight the intricate cross-talk between desmosomal proteins mutations and signaling pathways involved in the regulation of epicardial cell fate transition. We further emphasize that the consequences of desmosome deficiency differ between the embryonic and adult heart leading to enhanced erythropoiesis during heart development and enhanced fibrogenesis in the mature heart. We suggest that triggering epi-/endocardial cells and fibroblasts that are in different “states” involve the same pathways but lead to different pathological outcomes. Understanding the details of the different responses must be considered when developing interventions and therapeutic strategies.

## 1. Cardiogenesis and Mechanical Cues

Cardiogenesis is the orchestrated act of cell proliferation, differentiation, and migration that results in the formation of a resilient and durable contractile organ. The uninterrupted rhythmic contractions and the specific arrangement of cardiomyocytes and non-cardiomyocytes are the essential pillars of cardiac function. Deviation from cardiac cell identity and the canonical cell arrangement therefore not only alters cardiac morphogenesis but also impairs heart function. Our knowledge about the key genes that drive cardiogenesis comes from decades of animal studies [1]. It is increasingly recognized that the genetic profile and signaling molecules are not the only regulators of cardiac cell biology, but that mechanical cues also play a definitive role [2]. For example, the stiffness of the extracellular matrix modulates signaling in cardiomyocytes during development and disease [3,4]. Such multifactorial pathogenesis has been discussed in various cardiomyopathies including the still poorly understood genetic heart disease arrhythmogenic cardiomyopathy (AC).

Fifty percent of AC cases have been linked to mutations in genes that contribute to the formation of desmosomes, which are prominent cell–cell adhesion sites between cardiomyocytes [5,6]. Disease hallmarks are arrhythmia, loss of cardiomyocytes, and progressive formation of fibro–fatty structures, which lead to impairment of heart function and ultimately to heart failure [7]. Several transgenic animal models have been created to study AC pathogenesis [6]. The emerging insights categorize AC as a multifaceted disease involving disruption of mechanical and signaling pathways and a surge of immune responses [8]. This paper does not intend to provide a comprehensive review of AC pathogenesis and its clinical outcomes. Readers are referred to recent excellent reviews [9,10,11,12]. Instead, we will focus on the much less investigated and poorly understood functions of desmosomes in embryogenesis.

Desmosomal proteins are indispensable for early embryogenesis [13,14,15] and cardiogenesis [16,17]. Desmosomal proteins are enriched in intercalated discs of cardiomyocytes, but have been detected at low levels also in non-cardiomyocytes such as epicardial cells [18,19,20] and cardiac mesenchymal cells [21,22,23]. Importantly, cardiac mesenchymal cells also express desmosomal proteins [24] and desmosomal protein deficiency can enhance differentiation of these cells into fibrous or adipose tissue. In a recent study, a role of desmosomes has been suggested in regulation of cardiac mesenchymal cell fate by direct modulation of Ca^2+^ signaling at the level of gene expression [25].

During the last decades, research in the AC field was dominated by investigating the role of desmosomal proteins in adult cardiomyocytes [8,26]. It, however, remains to be explored whether desmosomal deficiency triggers “primary events” for the renewal or differentiation of cardiac progenitors both in the developing and mature heart. Of note, molecular pathways that are activated during embryonic development can be reactivated in pathological conditions [27]. This review focuses on the role of desmosomal proteins in cardial and epicardial morphogenesis. We begin with an overview of desmosomal proteins and structural differences between embryonic and adult hearts. This is followed by describing the development of cardiac progenitors and the formation of junctions in embryonic hearts. We further summarize current knowledge about the cross-talk between desmosomes and signaling pathways that mediate epicardial cell fate transition. In conclusion, we present a model whereby desmosomal deficiency manifests in different phenotypes depending on the pre-existing status of non-cardiomyocytes.

## 2. Molecular Structure of Desmosomes

**Figure 1:**
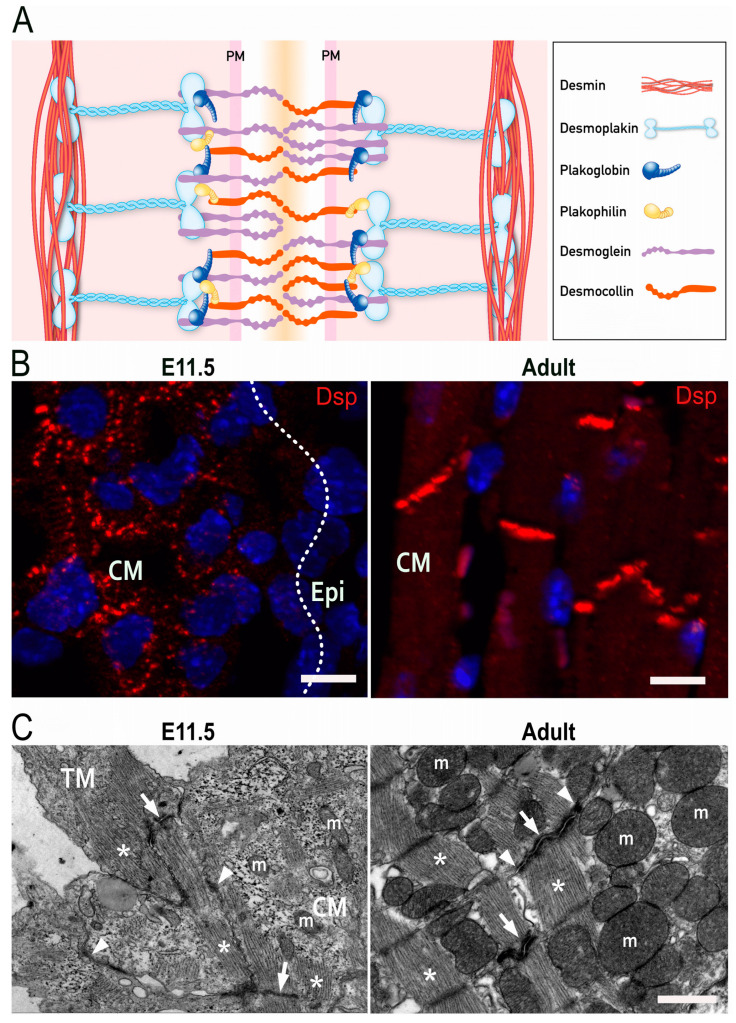
Desmosomes in the heart. (**A**) Scheme of the molecular desmosome structure. PM: plasma membrane. (**B**) Fluorescence microscopy detecting desmoplakin (Dsp; red) and nuclei (blue) in embryonic (**left**) and adult murine heart (**right**). CM: compact myocardium, Epi: Epicardium. Scale bars: 20 μm. (**C**) Electron micrographs of cardiomyocytes in embryonic (**left**) and adult murine hearts (**right**). Asterisks point to sarcomeres, arrows to actin-anchoring adherens junctions, and arrowheads to desmin-anchoring desmosomes. TM: trabecular myocardium; CM: compact myocardium; m, mitochondrion. Scale bar: 1 μm.

Desmosomes are specialized structures, which support the physical stability and integrity of epithelial and heart tissue. Cardiac desmosomes are formed by clustering of the Ca^2+^-dependent adhesion molecules (cadherins) desmoglein 2 (Dsg2) and desmocollin 2 (Dsc2) in the plasma membrane. They interact with each other via their extracellular domains to link neighboring cells. Intracellularly, the clustered desmosomal cadherins are connected to plakophilin 2 (Pkp2) and plakoglobin (PG), both of which contain multiple copies of the 42 amino acid-long armadillo repeat. They serve not only as structural linkers in desmosomes but fulfill additional cellular functions impacting adherens junctions, cytoskeletal organization, and gene transcription. The functions of the large cytolinker desmoplakin (Dsp), on the other hand, are much more restricted to desmosomes. Dsp is essential for the linkage between the clustered desmosomal cadherins with their associated armadillo-repeat proteins and the intermediate filament cytoskeleton, which consists of desmin polypeptides in cardiomyocytes (Figure 1A) [28,29]. Although the molecular composition of desmosomes is the same in the embryonic and adult heart, the arrangement of desmosomes differs. A distinctive characteristic of mammalian adult cardiomyocytes is their maturation process, which is initiated postnatally. Round-shaped embryonic cardiomyocytes initially form independent adherens junctions and desmosomes with neighboring cells throughout their entire borders. Concurrent with myofibril elongation and organization, desmosomes and adherens junctions concentrate at the apical surfaces of cardiomyocytes where they serve important mechanical functions [30]. Postnatally, the junctions amalgamate forming hybrid junctions that are composed of tightly integrated desmosomes and adherens junctions, which are in close apposition to gap junctions and membrane channels. This supercomplex has been referred to as area composita and connexome [31,32]. It was suggested that the maturation phase is essential to ensure life-long contraction of cardiomyocytes [31,33].

## 3. Cardiogenesis 

### 3.1. Contribution of Different Heart Fields 

Lineage tracing and anatomical studies revealed that Mesp1^+^ cardiac progenitors appear in the anterior splanchnic mesoderm layer at the lateral sides of the primitive streak [34]. The cardiac progenitor cells are arranged in two different heart fields, i.e., the first heart field (FHF) and the second heart field (SHF). They develop in a temporally and spatially distinct manner and participate in the formation of discrete parts of the heart [35], each with a unique gene profile [36,37]. The FHF comprises cardiac progenitors, which appear first and differentiate rapidly. These fast-differentiating cells express the transcription factors NKX2-5, Tbx5, Hand1, and GATA4 as well as the chromatin regulatory factor BAF60c. Together, these factors drive the expression of sarcomeric proteins [38]. Morphologically, the cells in the lateral regions of the FHF migrate toward the midline and form a tubular heart at embryonic day (E) 8 in mice and during the third week of gestation in humans. Slowly differentiating but rapidly proliferating cardiac progenitors appear posterior to the FHF to form the SHF. They express the transcription factors Isl-1 and TBX-1 [39,40,41]. Following heart tube formation, the SHF cells migrate into the heart tube from the atrial and venous poles. The coordinated movement of SHF and FHF cells is facilitated via receptor-ligand interaction and leads to the elongation of the heart tube and the formation of the right ventricle and outflow tract [42].

The lumen of the heart is lined by endocardial cells, which are a unique type of endothelial cells, both in terms of their origin and differentiation capacities [43]. Endocardial cells appear at the same time as cardiac progenitors with whom they share a common origin [44]. The endocardial and myocardial cell layers are separated by an extracellular matrix, which is composed of hyaluronic acid, fibronectin, collagen IV, and proteoglycans [45,46,47,48]. Endocardial cells can undergo endothelial-to-mesenchymal transition and transform into mesenchymal endocardial cushion cells, which subsequently remodel to form cardiac valves and separate the outflow track into the pulmonary artery and aorta. Endocardial cells exhibit remarkable plasticity differentiating into various lineages including endothelial cells of the capillary network, adipocytes, fibroblasts, and hematopoietic cells [44,49].

At mid-gestation (E10.5), two types of cardiomyocytes are present in the ventricular myocardium. The majority (86%) are immature cardiomyocytes, which are primarily located in the compact myocardium [50]. They have a spherical shape and contain little cytoplasm with loosely arranged myofilaments. The remaining cardiomyocytes (14%) are elongated and have regularly arranged sarcomeres [50]. They are generated from the compact myocardium by proliferation and delamination of cells and make up the trabecular myocardium [51]. Adherens and desmosomal junctions are present in both cardiomyocyte types. But the spherical-shaped myocytes contain intercellular junctions along all sides whereas the trabecular myocytes restrict the junctions to intercalated discs for the most part (Figure 1B,C).

### 3.2. Development of Epicardium and Epicardial-Derived Cells

Epicardial cells emerge from cell clusters that are referred to as the proepicardium (PE). The PE is located close to the liver primordium and sinus venosus. PE cells migrate toward the looped heart tube around E9.5, attach to the myocardium, and form the epicardial cell layer [52]. Prior to the attachment of epicardial cells to the myocardium at E9.5, the outer layer of cardiomyocytes is covered by a thin and patchy layer of fibronectin, laminin, and collagen IV, generating a basement membrane-like structure [53]. Epicardial cells initially contact myocytes directly through their α4 integrin receptor (CD49d), which binds VCAM-1 on adjacent cardiomyocytes [54]. Later, an extracellular matrix builds up between both cell layers. After formation of the epicardial layer, around E12 some epicardial cells undergo epithelial to mesenchymal transition (EMT), migrate into the sub-epicardial space and subsequently into the myocardium, where they can differentiate into fibroblasts, endothelial cells, and the smooth muscle cells surrounding arteries [55,56] as well as into mesenchymal stem cells [57].

At the same time, paracrine communication between epicardial and myocardial cells promotes myocardial growth [58]. In accordance, co-culture of embryonic epicardium-derived cells enhances the proliferation, maturation, and alignment of cardiomyocytes in vitro. This cross-talk involves increased expression of Cx43, N-cadherin, focal adhesion kinase, and sarcoplasmic reticulum Ca^2+^ ATPase [59]. Similarly, the promotion of structural and metabolic maturation of cardiomyocytes has been observed in co-cultures of cardiac fibroblasts (the derivatives of epicardial cells) with cardiomyocytes [60].

## 4. Development of Intercellular Junctions in Embryonic Cardiomyocytes

N-cadherin is the main cadherin of classical adherens junctions that are formed in cardiac progenitors as they appear in the cardiac crescent [61,62]. Immature and spherical cardiomyocytes establish multiple contacts with neighboring cells through N-cadherin-based junctions and maintain them as they are required for cardiomyocyte differentiation and organization [30,63,64,65]. During cardiogenesis, the localization of N-cadherins to intercalated discs is closely followed by the appearance of desmosomes [66]. The formation of adherens junctions is a prerequisite for desmosome formation. Loss of N-cadherin, therefore, destabilizes intercalated discs and desmosomes in adult cardiomyocytes [67]. It is even more detrimental during cardiogenesis inducing the formation of a disorganized myocardium with adhesion-deficient cardiomyocytes, reduced trabeculation, loss of cell polarity, and outward migration of cardiomyocytes to the pericardial cavity [51,68].

After the establishment of adherens junctions, the spherical cardiomyocytes of the compact myocardium establish additional contact points by forming desmosomal adhesions with neighboring cardiomyocytes. Desmosomal proteins such as Pkp2 and Dsp can be identified in cardiomyocytes as early as embryonic day 9.5 [17,62]. This goes along with prominent shape changes from spheroidal to elongated. After the appearance of desmosomes, gap junctions are formed and expanded in the plasma membrane [62]. In mature cardiomyocytes of the adult, desmosomes are clustered together with other junctions in the intercalated disc region leaving the lateral membranes desmosome-free.

In the following section, we will summarize the evidence, which indicates that a deficiency of desmosomal proteins can alter the fate of epicardial cells and will describe pathways that may play a role in converting this fate.

## 5. The Impact of Desmosomal Proteins on Cardiac Morphogenesis

Of all desmosomal proteins, Dsp and Dsg2 were found to be essential for early embryogenesis [13,69]. In particular, Dsp was identified as a common factor mediating both reprograming and regeneration, and its deficiency delayed in vitro reprogramming [70]. Rescue of *Dsp* mutation in extraembryonic tissue results in the progression of embryonic development up to E10, but then the mutants show drastic abnormalities in the organization of the myocardium with reduced mass in spite of unaltered apoptosis levels [16]. Subsequent studies revealed the importance of desmosomes for heart morphogenesis in several zebrafish and mouse models (Table 1; [16,17,71,72,73,74]).

Mutations in *Jup* (gene encoding PG) and *Pkp2* induce similar cardiogenesis abnormalities [74,76,77]. The hallmark of the *Jup^−/^^−^* phenotype is edema with the appearance of blood cells in the pericardial cavity along with reduced cell mass in the compact and trabecular myocardium. Despite normal development up to E9.5, *Jup^−/^^−^,* embryos die between E10.5 and E12.5. This time window corresponds to myocardial growth, the formation of epicardial cells, and epicardial to mesenchymal transition—events that are regulated by cross-talk between myocardial and epicardial cells. Similarly, Pkp2 expression is indispensable for heart development in zebrafish and mice [74,78,79]. Immunostaining of *Pkp2* null mice showed perturbed localization of desmosomal proteins in the myocardium [17], suggesting a key role of Pkp2 in stabilizing desmosome structure.

Despite the inability to document a rupture in *Pkp2* or *Jup* mutants, accumulating blood in the pericardial cavity was interpreted as leakage of blood due to weak cardiomyocyte junctions [17,74]. Our detailed histological assessment of embryonic hearts with *Dsg2* mutation suggests that rupture is not the cause of pericardial blood cell accumulation in most instances [72]. The analyses revealed that desmosome deficiency does not result in myocyte rupture or leakiness but that the accumulating blood in the pericardial cavity is caused by differentiation of endocardial/epicardial cells into hematopoietic Runx1^+^ cells, which excessively proliferate and subsequently transmigrate into the pericardial cavity [72]. Interestingly, the loss of cuboidal endothelial cell morphology and reduction of cell junctions between endocardial cells have also been observed in zebrafish treated with *Jup* morpholinos [73]. The reported blood-filled pericardial cavities in *Dsg2*, *Pkp2*, and *Jup* mutants suggest that the transition of epi-/endocardial cells to a hemogenic fate may be a common consequence of perturbed desmosome formation during cardiogenesis. Whether this transition of epi-/endocardial fate is a consequence of altered myocardium integrity or a direct consequence of desmosome deficiency in epicardial cells remains to be clarified.

## 6. Epithelial to Mesenchymal Transition in Desmosome Deficient Models

A wide spectrum of signaling molecules including the TGF-β superfamily, FGF, Notch, and Wnt mediate the cross-talk of epicardial and myocardial cells and prompt EMT (comprehensive recent reviews in [27,80,81]). Here, we will focus on activators of EMT that have been specifically associated with desmosome deficiency (Table 2). Furthermore, we will describe the divergent outcomes of desmosome deficiency/EMT between embryonic and adult hearts.

### 6.1. Desmosomes Communicate with Gap Junctions in the Regulation of Epicardial EMT

The interaction between desmosomes and the gap junction protein connexin 43 (Cx43) plays a role in maintaining the stability of plasma membrane-bound Cx43 in cardiomyocytes and epicardium of neonatal rats, albeit through different mechanisms [83,85]. It was observed that reducing Pkp2 levels using siRNA leads to a decrease in Cx43 plasma membrane localization [83]. In cardiomyocytes, super-resolution microscopy and proximity ligation assays demonstrated the localization of Pkp2 in Cx43-containing gap junction plaques indicating that Pkp2 has a direct role in Cx43 trafficking [86]. Furthermore, Dsp stabilizes Cx43 in the plasma membrane by inhibiting the activation of ERK1/2-MAPK and the phosphorylation of S278/282 residues in Cx43. This inhibition prevents clathrin-mediated internalization of Cx43, subsequently averting its lysosomal degradation [85].

During cardiogenesis, the loss of Cx43 results in reduced epicardial adhesion to the extracellular matrix as indicated by diminished ZO-1 immunostaining and smaller sized focal adhesions [87]. Morphologically, this manifests as detachment of epicardium from the myocardium, referred to as a “blistering phenotype” [87]. Mechanistically, reduction of Cx43 in epicardial cells is associated with alteration in actin cytoskeletal organization, increased migration speed, but loss of migration directionality, all of which reflects impaired EMT [87]. The in vivo consequence of impaired EMT in Cx43 deficiency is the abnormal development of coronary arteries in mice [88].

Numerous studies documented the cross-talk between Cx43 and the TGF-β signaling pathway, which includes the modulation of SMAD4 nuclear localization [89] as well as signaling involving Snail-1 [90,91,92]. However, some of these reports present conflicting results. For instance, blocking Cx43 in osteoblasts and retinal pigment epithelial cells inhibits TGF-β2-mediated EMT [93,94], while Cx43 siRNA leads to nuclear localization of SMAD-4 and activation of TGF-β targets in HL-1 cells [89]. Further exploration is required to understand the impact of desmosome deficiency on gap junction stability and activation of downstream pathways that influence the propensity of epicardial cells to undergo EMT in embryonic hearts.

### 6.2. Desmosome-TGF-β Cross-Talk in the Regulation of Epicardium Development

TGF-β signaling is one of the key drivers of cardiogenesis, and it is intriguing to explore whether desmosomal proteins regulate TGF-β signaling during cardiogenesis. TGF-β signaling is a well-recognized pathway in the regulation of EMT during development [95]. Given the intricate nature of the upstream activators of TGF-β signaling and its downstream targets, uncovering the potential interaction between desmosomes and regulators of TGF-β signaling is a nontrivial undertaking. But multiple pieces of evidence point to desmosome-TGF-β cross-talk. A deficiency of Pkp2 in cultured neonatal cardiomyocytes activates TGF-β1 expression via activation of p38 MAP kinase, leading to the expression of profibrotic genes [84]. In two murine AC disease models with constitutive or myocardial-restricted Dsg2 deficiency *Tgf-β mRNA* isoforms were upregulated [82]. In another AC disease model caused by adhesion-deficient Dsg2 activation of the profibrotic TGF-β pathway was reported and linked to the enrichment of ITG αV/β6 in intercalated discs [75]. Interestingly, epicardium-specific depletion of Dsp was shown to be sufficient to induce activation of the FGF2 and TGF-β1 pathways [21]. Altered TGF-β signaling in endothelial cells is most likely irrelevant since desmosomal proteins are not expressed in endothelial cells. Nevertheless, there is considerable ambiguity about the involvement of endocardial/cardiac endothelial cells in desmosome-deficient pathogenesis.

The previous literature, which reported activation of TGF-β signaling in epicardial-derived cells [21] is particularly interesting with regard to the observed expansion of blood clusters in embryonic *Dsg*- and *Pkp*-mutant murine myocardium [17,72,76]. Another complementary result is the expansion of blood cells in the pericardium of embryos with *VCAM-1* null mutation [54]. In accordance, the null mutation of the myocardial VCAM1 ligand integrin β4 (ITG-β4) results in a similar phenotype [96]. VCAM-1 antagonizes TGF-β stimulated weakening of intercellular junctions via modulation of stress fibers and diminishing Rho activity [97]. Therefore, the loss of epicardial cell attachment and expansion of blood cells in the pericardium can be explained by the activation of TGF-β signaling in the absence of VCAM-1 and ITG-β4. In epi/endocardial cells, TGF-β can induce EMT through multiple downstream signaling pathways involving phosphatidylinositol-3 kinase [98], wnt/β-catenin signaling [99], Rho [100], SMAD signaling [101], and the Jagged-1/Notch pathway [102,103]. Notably, epicardial activation of Notch signaling can induce subepicardial expansion of erythrocytes (known as epicardial blistering) in embryonic hearts [104]—a phenotype that is similar to that in *Dsg2* mutant embryos [72]. Another significant study focused on the generation of human *PKP2* null iPSC-derived epicardial cells. This study revealed that the long-term consequence of desmosome deficiency in epicardial cells includes the enrichment of mesenchymal markers such as CD73 and CD29. Additionally, there is a loss of Wt-1 expression, an elevation of markers associated with adipose and connective tissue, and activation of the transcription factor TFAP2A, which mediates EMT [22]. These findings are further supported by the validation of TFAP2A expression in cardiac biopsies of AC patients [22]. Importantly and in line with our argument, TFAP2A can activate TGF-β mediated EMT [105,106].

### 6.3. Desmosomes and Modulation of YAP/TAZ Signaling

Another critical pathway involved in heart development and cardiomyopathy is the YAP/TAZ pathway, which has been extensively reviewed (e.g., [107]). This pathway is activated upon disruption of cell polarity, loss of cell–cell contacts, mechanical stress, and increased extracellular matrix stiffness [108]. Similar situations are encountered in desmosome deficiency. In accordance, altered YAP signaling has been documented in AC animal models [109,110]. But, there exists still a considerable knowledge gap linking the YAP/TAZ pathway with desmosomal adhesion during cardiogenesis. Analyses conducted in different systems suggest that Cx43 expression counteracts YAP signaling. Specifically, the downregulation of Cx43 in astrocytes facilitated YAP nuclear translocation and regulated mesenchymal transition [111]. Moreover, the YAP/TAZ pathway has been found to converge with SMAD-mediated TGF-β signaling [112,113].

## 7. Conclusions and Future Directions

**Figure 2:**
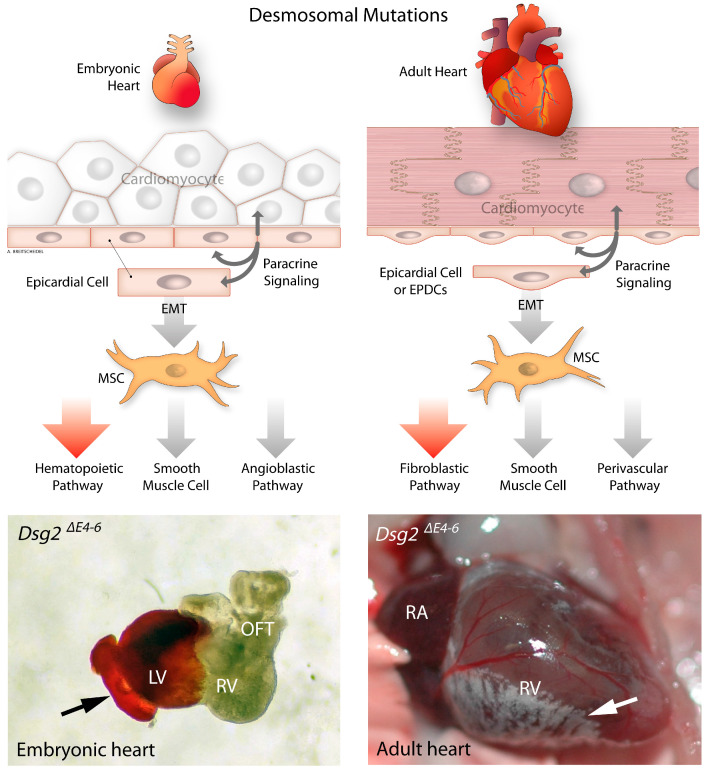
The impact of desmosomal mutation (*Dsg2*^Δ*E4–6*^) on the developing and mature murine heart. The scheme at left indicates that desmosome deficiency leads to remodeling of the embryonic heart, resulting in an expansion of hematopoietic stem cells and erythrocytes. The image below shows the dorsal side of an E11.5 heart with an excessive expansion of erythrocytes that populate the left ventricle and spread out to the pericardial side of the heart (arrow). The scheme at right shows an adult heart, which responds to desmoglein 2 mutation by cardiac remodeling resulting in the expansion of fibrotic cells. The photograph below depicts these changes in the right ventricle (arrow). OFT: outflow tract; RV: right ventricle; LV: left ventricle; RA: right atrium; EPDCs: Epicardial derived cells.

Taken together, we conclude that the combination of mechanical cues and signaling factors provides a microenvironment that promotes the escape of epicardial cells from their restricted fate. In embryonic hearts, desmosome deficiency-induced EMT leads to unrestricted expansion of hematopoietic stem cells [72]. Conversely, in adult hearts, the same mutation activates profibrotic pathways and the expansion of fibroblasts (Figure 2) [114]. We, therefore, suggest that the impact of desmosome deficiency in the heart depends primarily on the intrinsic, development-specific potentials of cells encountered in the heart including, besides cardiomyocytes, epi-, and endocardial cells. 

## Figures and Tables

**Table 1:** Desmosomal protein involvement in cardiogenesis.

		Cardiogenesis Phenotype									
Mutation	Organism	Edema	Pericardial Blood	Hypoplastic Myocard	Defective Contraction	Disrupted Myocardial Patterning	Defective Intercellular Adhesion	Rupture	Perturbed Endocardial Differentiation	Reduced Desmosomal Plaque	Refs.
*Dsc2^morpholino^*	Zebrafish	×			×					×	[71]
*Dsg2* ^Δ*E4–E6*^	Mouse	×	×	×		×	×	×	×		[72]
*Dsg2^W2A^*	Mouse	×	×								[75]
*Pkp2^morpholino^*	Zebrafish				×	×					[73]
*Pkp2^−/−^*	Mouse	×	×	×			×				[17]
*Jup^−/−^*	Mouse	×	×	×		×		×		×	[74,76]
*Dsp^−/− extraembryonal rescue^*	Mouse		×	×	×	×					[16]

**Table 2:** Interaction of desmosomal proteins with EMT pathways.

Mutation	Cell/Animal Model	Pathway	Refs.
*Dsg2^RNAi^*	Human pluripotent stem cells (hPSCs)	Inhibition of E-cadherin and elevation of Slug	[15]
*Dsg2* ^Δ*E4–E6*^ *Dsg2* ^Δ*E4–E5 myocardial induction*^	Adult murine heart	TGFβ and SRF signaling	[82]
*Dsg2^W2A^*	Adult murine heart	Integrin-αVβ6/TGF-β signaling	[75]
*Pkp2^RNAi^*	Neonatal rat ventricular myocytes (NRVMs) Epcardial-mesenchymal cells (EPDCs)	Reduction and redistribution of Cx43	[83]
*Pkp2^RNAi^*	Neonatal rat ventricular myocytes (NRVMs)	TGF-β1/p38 MAPK kinase signaling	[84]
*Pkp2^c.del2013c^*	Human-induced pluripotent stem cell- derived (hiPSC)-epicardial cells	Activation of TFAP2A	[22]
*Dsp^RNAi^*	Neonatal rat ventricular myocytes (NRVMs) Murine HL-1 atrial cardiomyocytes	ERK1/2-MAPK signaling Phosphorylation and degradation of Cx43	[85]
*Dsp^W/F epicardial induction^*	Adult Mouse Heart	FGF2 and TGF-β1 signaling	[21]

## Data Availability

Not applicable.
